# Correction: des Tombe, B., et al. Estimation of Temperature and Associated Uncertainty from Fiber-Optic Raman-Spectrum Distributed Temperature Sensing. *Sensors* 2020, *20*, 2235

**DOI:** 10.3390/s21030912

**Published:** 2021-01-29

**Authors:** Bas des Tombe, Bart Schilperoort, Mark Bakker

**Affiliations:** Water Resources Section, Faculty of Civil Engineering and Geosciences, Delft University of Technology, Stevinweg 1, 2628CN Delft, The Netherlands; B.Schilperoort@tudelft.nl (B.S.); mark.bakker@tudelft.nl (M.B.)

The authors wish to make the following two corrections to this paper [1]:

(1). In the original article, there was a mistake in Figure 6 as published. Standard uncertainty of the arithmetic mean should be corrected. The original Figure 6 appears below. 


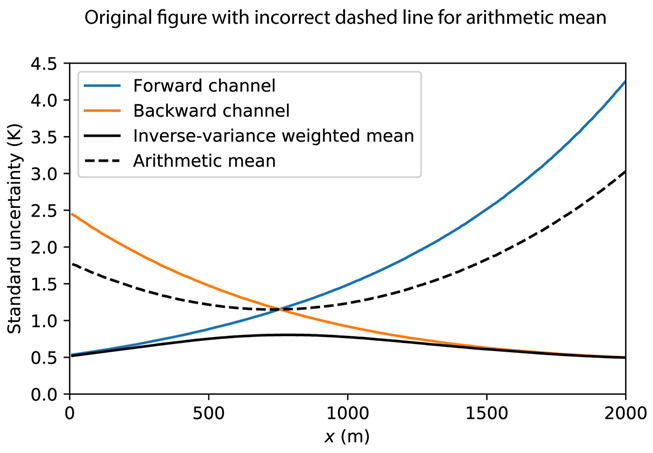


The corrected Figure 6 appears below.

**Figure 6:**
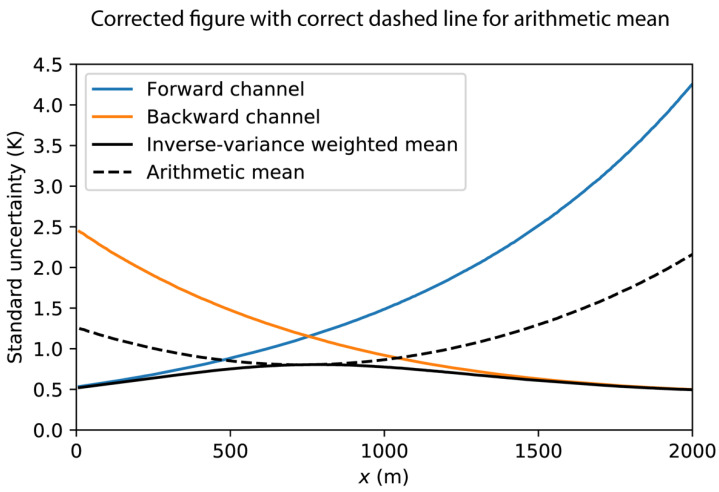
Synthetic example of the standard uncertainty of the estimated temperature using arithmetic mean and the inverse-variance weighted mean.

(2). Final sentence of Section 10.1 should be changed from “The standard uncertainty of the inverse-variance weighted mean is shown with the solid black line and is much smaller along the entire fiber” to "The standard uncertainty of the inverse-variance weighted mean is shown with the solid black line and is much smaller near the ends of the fiber. The standard uncertainty of the inverse-variance weighted mean is equal to that of the arithmetic mean where the standard uncertainty of the forward-channel measurements is equal to that of the backward-channel measurements”.

The authors apologize for any inconvenience caused and state that the scientific conclusions are unaffected. The original article has been updated.
