# Supplementary material for: Estimation of Temperature and Associated Uncertainty from Fiber-Optic Raman-Spectrum Distributed Temperature Sensing
Source: Sensors (Basel). 2020 Apr 15;20(8):2235. doi: 10.3390/s20082235 (PMC7218869; doi:10.3390/s20082235)
Supplement: Supplementary file 1 [file sensors-20-02235-s001.zip › Article_S1.pdf]

Article_S1

February 17, 2020

[1]:
```
import dask.array as da
import dtscalibration
import matplotlib.pyplot as plt
import numpy as np
import xarray as xr
```

```
/Users/bfdestombe/anaconda3/lib/python3.7/typing.py:845: FutureWarning: xarray
subclass DataStore should explicitly define __slots__
  super().__init_subclass__(*args, **kwargs)
```

[2]:
```
import sys; print('Python %s on %s' % (sys.version, sys.platform))
```

```
Python 3.7.5 (default, Oct 25 2019, 10:52:18)
[Clang 4.0.1 (tags/RELEASE_401/final)] on darwin
```

[3]:
```
!pip show dtscalibration
```

```
Name: dtscalibration
Version: 0.8.0
Summary: A Python package to load raw DTS files, perform a calibration, and plot
the result
Home-page: https://github.com/dtscalibration/python-dts-calibration
Author: Bas des Tombe, Bart Schilperoort
Author-email: bdestombe@gmail.com
License: BSD 3-Clause License
Location: /Users/bfdestombe/Projects/dts-calibration/python-dts-calibration-
dev/src
Requires: numpy, xarray, pyyaml, xmltodict, scipy, patsy, statsmodels, nbsphinx,
dask, toolz, matplotlib, netCDF4, pandas, cloudpickle
Required-by:
```

[4]:
```
!pip list
```

```
Package                       Version             Location
----------------------------- -------------------
-------------------------------------------------------------------------
alabaster                     0.7.12
appdirs                       1.4.3
```

appnope	0.1.0
attrs	19.1.0
Babel	2.7.0
backcall	0.1.0
black	19.10b0
bleach	3.1.0
bokeh	1.3.4
certifi	2019.9.11
cftime	1.0.3.4
chardet	3.0.4
click	6.7
cloudpickle	1.2.2
colorcet	2.0.1
cycler	0.10.0
dask	2.10.1
datashader	0.7.0
datashape	0.5.2
decorator	4.4.1
defusedxml	0.6.0
distributed	2.2.0
docopt	0.6.2
docutils	0.14
dtscalibration	0.8.0
/Users/bfdestombe/Projects/dts-calibration/python-dts-calibration-dev/src	
entrypoints	0.3
filelock	3.0.12
HeapDict	1.0.0
holoviews	1.12.1
hvplot	0.4.0
idna	2.8
imageio	2.5.0
imagesize	1.1.0
importlib-metadata	0.23
ipython	7.9.0
ipython-genutils	0.2.0
jedi	0.15.1
Jinja2	2.10.1
jsonschema	3.0.1
jupyter-core	4.5.0
kiwisolver	1.1.0
llvmlite	0.30.0
locket	0.2.0
Markdown	3.1.1
MarkupSafe	1.1.1
matplotlib	3.1.1
mistune	0.8.4
mkl-fft	1.0.14
mkl-random	1.1.0

mkl-service	2.3.0
more-itertools	7.2.0
mpmath	1.1.0
msgpack	0.6.1
multipledispatch	0.6.0
nbconvert	5.5.0
nbformat	4.4.0
nbsphinx	0.4.2
netCDF4	1.5.1.2
networkx	2.3
numba	0.46.0
numpy	1.18.1
packaging	19.0
pandas	0.24.2
pandocfilters	1.4.2
panel	0.6.2
param	1.9.1
parso	0.5.1
partd	1.0.0
pathspec	0.7.0
patsy	0.5.1
pexpect	4.7.0
pickleshare	0.7.5
Pillow	6.1.0
pip	19.3.1
pluggy	0.13.0
prompt-toolkit	2.0.10
psutil	5.6.3
ptyprocess	0.6.0
py	1.8.0
pyct	0.4.6
Pygments	2.4.2
pykwalify	1.7.0
pyparsing	2.4.2
PyQt5-sip	4.19.18
pyrsistent	0.15.3
python-dateutil	2.8.0
pytz	2019.3
pyviz-comms	0.7.2
PyWavelets	1.0.3
PyYAML	5.1.1
regex	2020.1.8
requests	2.22.0
ruamel.yaml	0.15.100
scikit-image	0.15.0
scipy	1.3.0
setuptools	41.6.0.post20191030
six	1.12.0

```
snowballstemmer                 1.9.0
sortedcontainers                2.1.0
Sphinx                          2.1.2
sphinxcontrib-applehelp         1.0.1
sphinxcontrib-devhelp           1.0.1
sphinxcontrib-htmlhelp          1.0.2
sphinxcontrib-jsmath            1.0.1
sphinxcontrib-qthelp            1.0.2
sphinxcontrib-serializinghtml   1.1.3
statsmodels                     0.10.0
tblib                           1.4.0
testpath                        0.3.1
toml                            0.10.0
toolz                           0.10.0
tornado                         6.0.3
tox                             3.14.0
traitlets                       4.3.3
typed-ast                       1.4.1
urllib3                         1.25.3
virtualenv                      16.7.7
wcwidth                         0.1.7
webencodings                    0.5.1
wheel                           0.33.6
xarray                          0.12.3
xmltodict                       0.12.0
xrviz                           0.1.1
yapf                            0.28.0
zict                            1.0.0
zipp                            0.6.0
```

[5]:
```
opendap_url = r"/Users/bfdestombe/Google Drive/PhD/research/DTS " \
              r"error/onedaynetcdf/calibrated1000x2sec.nc"
# opendap_url = r"https://opendap.tudelft.nl/thredds/dodsC/data2/uuid/
↪71b5c3c2-4105-4f4f-bd1e-d7c56732a665/calibrated1000x2sec.nc"
```

[6]:
```
sections = {
    'probe1Temperature':  [slice(7.5, 17.), slice(70., 80.)],  # cold bath
    'probe2Temperature':  [slice(24., 34.), slice(85., 95.)],  # warm bath
    'external_probe_dts': [slice(39., 48.)]
    }
calibration_sections = {
    'probe1Temperature': [slice(7.5, 17.)],  # cold bath
    'probe2Temperature': [slice(24., 34.)],  # warm bath
    }
valid_measurement_range = slice(0.0, 97.0)   # only consider parts of the fiber
```

[7]:
```
ds = dtscalibration.open_datastore(opendap_url, load_in_memory=True)

ds = ds.sel(x=valid_measurement_range).isel(
    x=slice(None, None, 2))  # Only use every other measurement, to minimize␣
↪spatial correlation
```

1 Perform the calibration

[8]:
```
ds.sections = calibration_sections
```

[9]:
```
st_var, st_resid = ds.variance_stokes(st_label='ST')
ast_var, ast_resid = ds.variance_stokes(st_label='AST')
rst_var, rst_resid = ds.variance_stokes(st_label='REV-ST')
rast_var, rast_resid = ds.variance_stokes(st_label='REV-AST')
```

[10]:
```
nmc = 100  # Increase to 10000, but computation time increases quickly.
ds.calibration_double_ended(
    st_var=st_var,
    ast_var=ast_var,
    rst_var=rst_var,
    rast_var=rast_var,
    store_tmpw='TMPW',
    method='wls',
    solver='sparse',
    tmpw_mc_size=nmc)
ds = ds.compute()  # Easily fits in everyone's computer memory. To speed up␣
↪computation.
```

[11]:
```
# For the statistics use all sections
ds.sections = sections

ds.conf_int_double_ended(
    st_var=st_var,
    ast_var=ast_var,
    rst_var=rst_var,
    rast_var=rast_var,
    conf_ints=[2.5, 97.5],
    ci_avg_time_flag=False,
    ci_avg_x_flag=True,
    store_tempvar='_var_x',
    remove_mc_set_flag=True,
    mc_sample_size=nmc,  # <- choose a much larger sample size)
    var_only_sections=True)
```

[12]:
```
ds['TMPW'] = ds['TMPW'].compute()  # We need this one for many calculations
```

```python
# For the statistics use all sections
ds.sections = sections

# Construct full array with measurements from external temperature sensor
# by broadcasting the reference temperatures.
tmp_ref_arr = ds.ufunc_per_section(
    label='ST',
    ref_temp_broadcasted=True,
    calc_per='all')
ix_resid = ds.ufunc_per_section(
    x_indices=True,
    calc_per='all')
tmp_ref_sorted = np.full(
    shape=ds.ST.shape, fill_value=np.nan)
tmp_ref_sorted[ix_resid, :] = tmp_ref_arr
tmp_ref_da = xr.DataArray(
    data=tmp_ref_sorted,
    coords=ds.ST.coords)
```

[13]:
```python
q = ds['TMPW'] - tmp_ref_da.values

ds['TMPW_mean_unc_time'] = q.mean(dim='hours').compute()
ds['TMPW_mean_unc_x'] = q.mean(dim='x').compute()

ds['TMPW_std_unc_time'] = q.std(dim='hours').compute()
ds['TMPW_std_unc_x'] = q.std(dim='x').compute()
```

```
/Users/bfdestombe/anaconda3/lib/python3.7/site-
packages/xarray/core/nanops.py:140: RuntimeWarning: Mean of empty slice
  return np.nanmean(a, axis=axis, dtype=dtype)
```

[14]:
```python
ds.conf_int_double_ended(
    st_var=st_var,
    ast_var=ast_var,
    rst_var=rst_var,
    rast_var=rast_var,
    conf_ints=[2.5, 97.5],
    ci_avg_time_flag=False,
    ci_avg_x_flag=False,
    mc_sample_size=nmc)  # <- choose a much larger sample size)
```

[15]:
```python
# ds.to_netcdf(r"paper_sensors.nc", encoding={})
```

2 Table 2

[16]:
```
print('The values may differ, depending on the number of Monte Carlo samples␣
 ↪used (nmc).')

# ds['in_ci'] contains a boolean array whether the reference temperatures fall␣
 ↪within the confidence intervals
ds['in_ci'] = ds.in_confidence_interval('TMPW_MC', [2.5, 97.5])

f = lambda x: np.sum(x) / np.size(x) * 100
poster_table = da.compute(
    ds.ufunc_per_section(label='in_ci', func=f, calc_per='stretch'))
print('Per reference section: \n\t', poster_table)
poster_table = da.compute(
    ds.ufunc_per_section(label='in_ci', func=f, calc_per='all'))
print('\n\nFor all reference section: \n\t', poster_table)
```

```
The values may differ, depending on the number of Monte Carlo samples used
(nmc).

/Users/bfdestombe/anaconda3/lib/python3.7/site-packages/dask/array/core.py:1328:
FutureWarning: The `numpy.size` function is not implemented by Dask array. You
may want to use the da.map_blocks function or something similar to silence this
warning. Your code may stop working in a future release.
  FutureWarning,
/Users/bfdestombe/anaconda3/lib/python3.7/site-packages/dask/core.py:119:
RuntimeWarning: invalid value encountered in less_equal
  return func(*args2)
/Users/bfdestombe/anaconda3/lib/python3.7/site-packages/dask/core.py:119:
RuntimeWarning: invalid value encountered in greater_equal
  return func(*args2)
/Users/bfdestombe/anaconda3/lib/python3.7/site-packages/dask/array/core.py:1328:
FutureWarning: The `numpy.size` function is not implemented by Dask array. You
may want to use the da.map_blocks function or something similar to silence this
warning. Your code may stop working in a future release.
  FutureWarning,
/Users/bfdestombe/anaconda3/lib/python3.7/site-packages/dask/core.py:119:
RuntimeWarning: invalid value encountered in less_equal
  return func(*args2)
/Users/bfdestombe/anaconda3/lib/python3.7/site-packages/dask/core.py:119:
RuntimeWarning: invalid value encountered in greater_equal
  return func(*args2)
/Users/bfdestombe/anaconda3/lib/python3.7/site-packages/dask/array/core.py:1328:
FutureWarning: The `numpy.size` function is not implemented by Dask array. You
may want to use the da.map_blocks function or something similar to silence this
warning. Your code may stop working in a future release.
  FutureWarning,
/Users/bfdestombe/anaconda3/lib/python3.7/site-packages/dask/core.py:119:
```

```
RuntimeWarning: invalid value encountered in less_equal
  return func(*args2)
/Users/bfdestombe/anaconda3/lib/python3.7/site-packages/dask/core.py:119:
RuntimeWarning: invalid value encountered in greater_equal
  return func(*args2)
/Users/bfdestombe/anaconda3/lib/python3.7/site-packages/dask/array/core.py:1328:
FutureWarning: The `numpy.size` function is not implemented by Dask array. You
may want to use the da.map_blocks function or something similar to silence this
warning. Your code may stop working in a future release.
  FutureWarning,
/Users/bfdestombe/anaconda3/lib/python3.7/site-packages/dask/core.py:119:
RuntimeWarning: invalid value encountered in less_equal
  return func(*args2)
/Users/bfdestombe/anaconda3/lib/python3.7/site-packages/dask/core.py:119:
RuntimeWarning: invalid value encountered in greater_equal
  return func(*args2)
/Users/bfdestombe/anaconda3/lib/python3.7/site-packages/dask/array/core.py:1328:
FutureWarning: The `numpy.size` function is not implemented by Dask array. You
may want to use the da.map_blocks function or something similar to silence this
warning. Your code may stop working in a future release.
  FutureWarning,
/Users/bfdestombe/anaconda3/lib/python3.7/site-packages/dask/core.py:119:
RuntimeWarning: invalid value encountered in less_equal
  return func(*args2)
/Users/bfdestombe/anaconda3/lib/python3.7/site-packages/dask/core.py:119:
RuntimeWarning: invalid value encountered in greater_equal
  return func(*args2)
/Users/bfdestombe/anaconda3/lib/python3.7/site-packages/dask/core.py:119:
RuntimeWarning: invalid value encountered in less_equal
  return func(*args2)
/Users/bfdestombe/anaconda3/lib/python3.7/site-packages/dask/core.py:119:
RuntimeWarning: invalid value encountered in greater_equal
  return func(*args2)
```

```
Per reference section:
         ({'external_probe_dts': [90.03714285714285], 'probe1Temperature':
[93.54864864864865, 92.67179487179487], 'probe2Temperature': [92.92564102564103,
92.03846153846153]},)
```

```
/Users/bfdestombe/anaconda3/lib/python3.7/site-packages/dask/array/core.py:1328:
FutureWarning: The `numpy.size` function is not implemented by Dask array. You
may want to use the da.map_blocks function or something similar to silence this
warning. Your code may stop working in a future release.
  FutureWarning,
/Users/bfdestombe/anaconda3/lib/python3.7/site-packages/dask/core.py:119:
RuntimeWarning: invalid value encountered in less_equal
  return func(*args2)
/Users/bfdestombe/anaconda3/lib/python3.7/site-packages/dask/core.py:119:
RuntimeWarning: invalid value encountered in greater_equal
```

```
  return func(*args2)
/Users/bfdestombe/anaconda3/lib/python3.7/site-packages/dask/core.py:119:
RuntimeWarning: invalid value encountered in less_equal
  return func(*args2)
/Users/bfdestombe/anaconda3/lib/python3.7/site-packages/dask/core.py:119:
RuntimeWarning: invalid value encountered in greater_equal
  return func(*args2)
```

```
For all reference section:
         (92.27724867724868,)
```

3 Figure 3. Confidence interval of the temperature at the first time step.

[18]:

```
print('The plots may slightly differ, depending on the number of Monte Carlo␣
 ↪samples used (nmc).\n' +
'Increasing the Monte Carlo sample size smoothens the edges of the confidence␣
 ↪interval.')

ds.sections = sections

sections_names = {
    'Cold 1':  [slice(7.5, 17.)],
    'Warm 1':  [slice(24., 34.)],
    'Ambient': [slice(40., 50.)],
    'Cold 2':  [slice(70., 80.)],
    'Warm 2':  [slice(85., 95.)]
    }
sections_names2 = {
    'Cold 1':  'Used for calibration',
    'Warm 1':  'Used for calibration',
    'Ambient': '',
    'Cold 2':  '',
    'Warm 2':  ''
    }

# Construct figure
it = 0
fig, axs = plt.subplots(2, 1, figsize=(10, 7), sharex=True,
                        gridspec_kw=dict(hspace=0.05, wspace=0.))

title = 'Estimated temperature and 95% confidence interval at time step 1'
fig.suptitle(title, y=0.91)

# subfig a
```

```
ax = axs[0]

dn = ds.TMPW_MC.isel(CI=0, hours=it).values
up = ds.TMPW_MC.isel(CI=1, hours=it).values
val = ds.TMPW.isel(hours=it).values
x = ds.x.values
ax.fill_between(x, dn, up, facecolor='C0', label='95% Confidence Interval',
                alpha=0.3)
ax.plot(x, val, linewidth=0.9, c='black', label='Estimated temperature')

ax.set_ylabel('temperature ($^\circ$C)')
ax.set_ylim([1.9, 20])
ax.legend(loc='upper left')

ylim_bot = 3.

for k, v in sections_names.items():
    for vi in v:
        ax.plot([vi.start, vi.stop], [ylim_bot, ylim_bot],
                linewidth=0.8,
                c='black')
        tbx, tby = (vi.start + vi.stop) / 2, ylim_bot
        ax.annotate(k,
                    xy=(tbx, tby),
                    ha='center',
                    va='bottom',
                    fontsize=8,
                    xytext=(0, 2),
                    textcoords='offset points')  # ,

sections_names2 = {
    'Cold 1':  'Used for calibration',
    'Warm 1':  'Used for calibration',
    'Ambient': '',
    'Cold 2':  '',
    'Warm 2':  ''
    }

for (k, v), (k2, v2) in zip(sections_names.items(), sections_names2.items()):
    for vi in v:
        tbx, tby = (vi.start + vi.stop) / 2, ylim_bot
        ax.annotate(v2,
                    xy=(tbx, tby),
                    ha='center',
                    va='top',
                    fontsize=8,
                    xytext=(0, -2),
```

```
                    textcoords='offset points')
# plt.show()

ds2 = dtscalibration.open_datastore(opendap_url,
                                    chunks={})
ds2.sections = {
    'probe1Temperature':  [slice(7.5, 17.), slice(70., 80.)],  # cold bath
    'probe2Temperature':  [slice(24., 34.), slice(85., 95.)],  # warm bath
    'external_probe_dts': [slice(39., 48.)]
    }
ds2['in_ci'] = ds2.in_confidence_interval('TMPW_MC', [2.5, 97.5])

poster_table = da.compute(
    ds2.ufunc_per_section(label='in_ci', func=np.sum, calc_per='stretch'))
print(poster_table)

# subfig b
ax = axs[1]
x = ds.x.values

ds_sel = ds.isel(hours=it)

tmpw = ds_sel.TMPW

tmp_ref_arr = ds.ufunc_per_section(label='ST',
                                   ref_temp_broadcasted=True,
                                   calc_per='all')
ix_resid = ds.ufunc_per_section(x_indices=True, calc_per='all')

tmp_ref_sorted = np.full(shape=ds.ST.shape, fill_value=np.nan)
tmp_ref_sorted[ix_resid, :] = tmp_ref_arr
tmp_ref_da = xr.DataArray(data=tmp_ref_sorted, coords=ds.ST.coords)
tmp_ref = tmp_ref_da.isel(hours=it)

meas = (tmpw - tmp_ref).values
up = (tmpw - ds_sel.TMPW_MC.isel(CI=0)).values
dn = (tmpw - ds_sel.TMPW_MC.isel(CI=1)).values

ax.fill_between(x, dn, up, label='95% Confidence Interval', alpha=0.3,
                facecolor='C0')
ax.axhline(0., c='black', linewidth=0.9)
out_mask = np.logical_or(meas >= up, meas <= dn)
in_mask = np.logical_or(meas < up, meas > dn)
ax.scatter(x[in_mask],
           meas[in_mask],
           marker='.',
           c='black',
```

```python
            label='Inside confidence interval')
ax.scatter(x[out_mask],
            meas[out_mask],
            marker='*',
            c='red',
            label='Outside confidence interval')

ax.legend(loc='upper left')
ax.set_ylim([-0.5, 0.5])

ylim_bot = ax.get_ylim()[0] + 0.06 * (ax.get_ylim()[1] - ax.get_ylim()[0])

for k, v in sections_names.items():
    for vi in v:
        ax.plot([vi.start, vi.stop], [ylim_bot, ylim_bot],
                linewidth=0.8,
                c='black')
        tbx, tby = (vi.start + vi.stop) / 2, ylim_bot
        ax.annotate(k,
                    xy=(tbx, tby),
                    ha='center',
                    va='bottom',
                    fontsize=8,
                    xytext=(0, 2),
                    textcoords='offset points')  # ,
        # bbox=dict(fc='white', alpha=0.4, color='none'))

for (k, v), (k2, v2) in zip(sections_names.items(), sections_names2.items()):
    for vi in v:
        tbx, tby = (vi.start + vi.stop) / 2, ylim_bot
        ax.annotate(v2,
                    xy=(tbx, tby),
                    ha='center',
                    va='top',
                    fontsize=8,
                    xytext=(0, -2),
                    textcoords='offset points')

ax.set_xlabel('$x$ (m)')
ax.set_ylabel('$T$ - $T_\mathsf{reference}$ ($^\circ$C)')
# fig.tight_layout()
fig.text(0.05, 0.86, 'a)', fontsize=14)
fig.text(0.05, 0.46, 'b)', fontsize=14)
plt.show()
```

The plots may slightly differ, depending on the number of Monte Carlo samples used (nmc).

Increasing the Monte Carlo sample size smoothens the edges of the confidence interval.

```
/Users/bfdestombe/anaconda3/lib/python3.7/site-packages/dask/core.py:119:
RuntimeWarning: invalid value encountered in less_equal
  return func(*args2)
/Users/bfdestombe/anaconda3/lib/python3.7/site-packages/dask/core.py:119:
RuntimeWarning: invalid value encountered in greater_equal
  return func(*args2)
```

```
({'external_probe_dts': [66519], 'probe1Temperature': [71538, 74090],
'probe2Temperature': [73639, 73029]},)
```

```
/Users/bfdestombe/anaconda3/lib/python3.7/site-
packages/ipykernel_launcher.py:118: RuntimeWarning: invalid value encountered in
greater_equal
/Users/bfdestombe/anaconda3/lib/python3.7/site-
packages/ipykernel_launcher.py:118: RuntimeWarning: invalid value encountered in
less_equal
/Users/bfdestombe/anaconda3/lib/python3.7/site-
packages/ipykernel_launcher.py:119: RuntimeWarning: invalid value encountered in
less
/Users/bfdestombe/anaconda3/lib/python3.7/site-
packages/ipykernel_launcher.py:119: RuntimeWarning: invalid value encountered in
greater
```

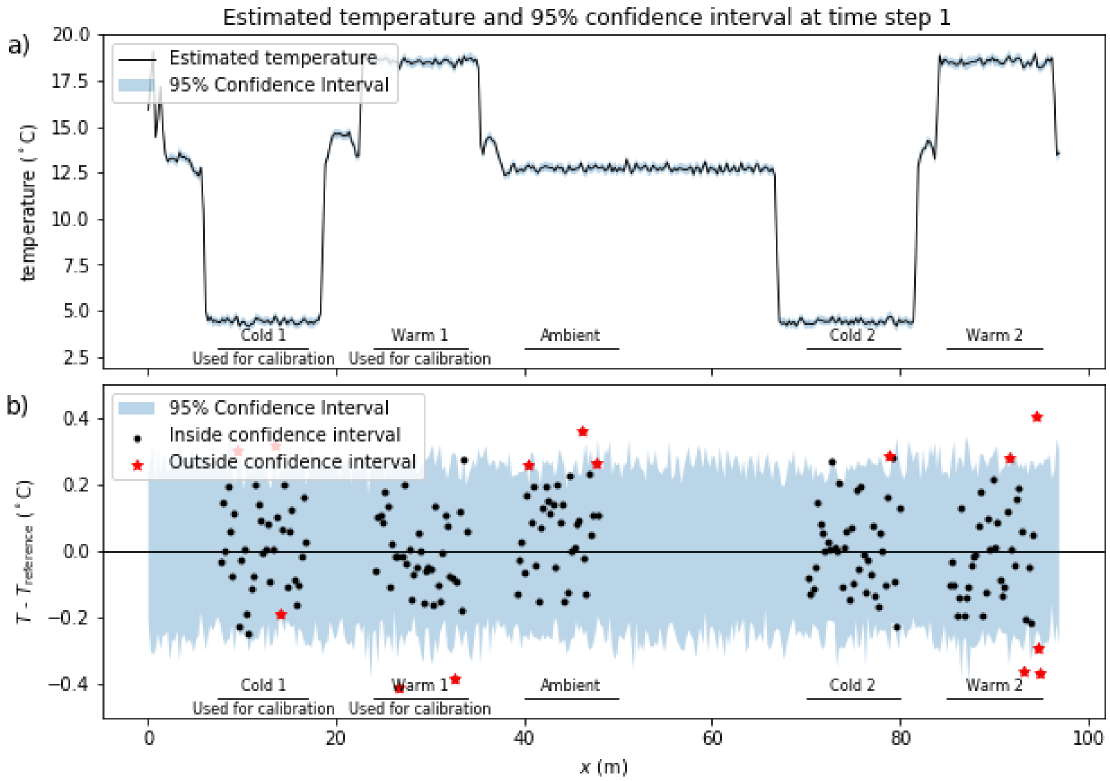

4 Figure 4. Spatial variation of the uncertainty.

[19]:

```
# spatial variation
fig, ax = plt.subplots(figsize=(10, 3))
ax.axhline(0., linewidth=0.8, c='black')
ax.axhspan(None, None, label='Standard uncertainty', facecolor='C0')
ax.axhspan(None, None, label='Mean difference', facecolor='C1')

# std
y_est = (ds.TMPW_MC_var_time**0.5).values
x_est = (ds.TMPW_MC_var_time**0.5).x.values
ax.plot(x_est, y_est, linewidth=3.5, c='white',
        alpha=0.5)
ax.plot(x_est, y_est, linewidth=2., c='white')
ax.plot(x_est, y_est, linewidth=0.8, c='black')
# ax.plot(x_est, y_est, linewidth=0.8, c='black', dashes=[7.5, 5])

y_err = ds.TMPW_std_unc_time.values
x_err = ds.TMPW_std_unc_time.x.values
ax.scatter(x_err, y_err, marker='x', s=9)

y_err = ds.TMPW_mean_unc_time.values
x_err = ds.TMPW_mean_unc_time.x.values

ax.scatter(x_err, y_err, marker='.')

# labels
sections_names = {
    'Cold 1':  [slice(7.5, 17.)],
    'Warm 1':  [slice(24., 34.)],
    'Ambient': [slice(40., 50.)],
    'Cold 2':  [slice(70., 80.)],
    'Warm 2':  [slice(85., 95.)]
    }
sections_names2 = {
    'Cold 1':  'Used for calibration',
    'Warm 1':  'Used for calibration',
    'Ambient': '',
    'Cold 2':  '',
    'Warm 2':  ''
    }
ylim_bot = -0.05
for k, v in sections_names.items():
    for vi in v:
        ax.plot([vi.start, vi.stop], [ylim_bot, ylim_bot],
```

```
                linewidth=0.8,
                c='black')
        tbx, tby = (vi.start + vi.stop) / 2, ylim_bot
        ax.annotate(k,
                    xy=(tbx, tby),
                    ha='center',
                    va='bottom',
                    fontsize=8,
                    xytext=(0, 2),
                    textcoords='offset points')  # ,
for (k, v), (k2, v2) in zip(sections_names.items(), sections_names2.items()):
    for vi in v:
        tbx, tby = (vi.start + vi.stop) / 2, ylim_bot
        ax.annotate(v2,
                    xy=(tbx, tby),
                    ha='center',
                    va='top',
                    fontsize=8,
                    xytext=(0, -2),
                    textcoords='offset points')

h_std_unc1 = ax.plot([], [], linewidth=4, c='black')
h_std = ax.scatter([], [], marker='x', s=18, color='C0')
h_avg = ax.scatter([], [], marker='.', s=80, color='C1')
ax.legend([h_std_unc1[0], h_std, h_avg],
          ['Standard uncertainty', 'Standard deviation of differences',
           'Mean of differences'],
          loc='center right', fontsize='small')
ax.set_xlabel('x (m)')
ax.set_ylabel('temperature ($^\circ$C)')
ax.set_ylim((-0.07, 0.16))
# fig.tight_layout()
fig.subplots_adjust(bottom=0.15)

print('The plots may slightly differ, depending on the number of Monte Carlo␣
 ↪samples used (nmc)')
```

```
The plots may slightly differ, depending on the number of Monte Carlo samples
used (nmc)
```

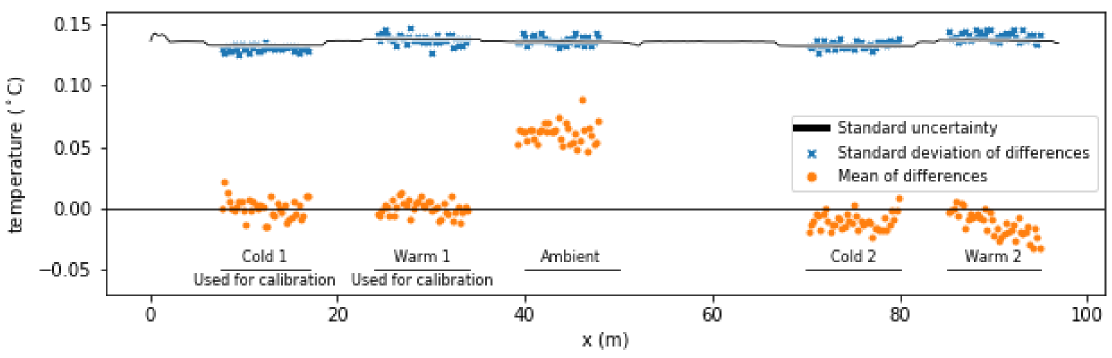

5 Figure 5. Temporal variation of the uncertainty.

[20]:

```
fig, ax = plt.subplots(figsize=(10, 3))
ax.axhline(0., linewidth=0.8, c='black')

# std
y_est = (ds.TMPW_MC_var_x**0.5).values
x_est = (ds.TMPW_MC_var_x**0.5).hours.values
ax.plot(x_est, y_est, linewidth=3.5, c='white', alpha=0.5)
ax.plot(x_est, y_est, linewidth=2., c='white')
ax.plot(x_est, y_est, linewidth=0.8, c='black')

y_err = ds.TMPW_std_unc_x.values
x_err = ds.TMPW_std_unc_x.hours.values
ax.scatter(x_err, y_err, marker='x', s=9, color='C0')


y_err = ds.TMPW_mean_unc_x.values
x_err = ds.TMPW_mean_unc_x.hours.values

ax.scatter(x_err, y_err, marker='.', color='C1')

ax.set_ylabel('temperature ($^\circ$C)')
ax.set_ylim((-0.05, 0.17))
ax.set_xlabel('time (hours)')

# legend
h_std_unc1 = ax.plot([], [], linewidth=4, c='black')
h_std = ax.scatter([], [], marker='x', s=18, color='C0')
h_avg = ax.scatter([], [], marker='.', s=80, color='C1')
ax.legend([h_std_unc1[0], h_std, h_avg],
          ['Standard uncertainty', 'Standard deviation of differences',
           'Mean of differences'],
```

```
            loc='center right', fontsize='small')

# fig.tight_layout()
fig.subplots_adjust(bottom=0.15)
```

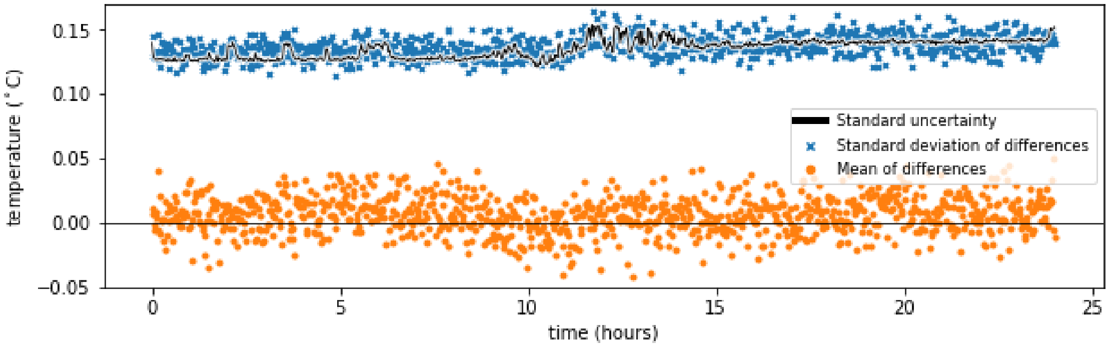

[ ]:

```
```
